# Differential Neuronal Development in iPSC‐Derived Neural Stem Cells From Monozygotic Twin Cases With Treatment‐Resistant Schizophrenia and Discordant Responses to Clozapine

**DOI:** 10.1002/npr2.70097

**Published:** 2026-03-22

**Authors:** Shotaro Kawano, Sayaka Katayama, Masaya Ogawa, Rei Endo, Naoto Ikeda, Yuuri Ikeuchi, Tomoki Mita, Hikari Takei, Nanaka Gotoda‐Nishimura, Daiki Miura, Hotaka Fukushima, Hitoshi Hashimoto, Ryota Hashimoto, Takanobu Nakazawa

**Affiliations:** ^1^ Laboratory of Molecular Biology, Department of Bioscience, Graduate School of Life Sciences Tokyo University of Agriculture Tokyo Japan; ^2^ Laboratory of Molecular Neuropharmacology, Graduate School of Pharmaceutical Sciences Osaka University Suita Japan; ^3^ Molecular Research Center for Children's Mental Development, United Graduate School of Child Development Osaka University, Kanazawa University, Hamamatsu University School of Medicine, Chiba University and University of Fukui Suita Japan; ^4^ D3 Center Osaka University Suita Japan; ^5^ Transdimensional Life Imaging Division Institute for Open and Transdisciplinary Research Initiatives, Osaka University Suita Japan; ^6^ Department of Molecular Pharmaceutical Science, Graduate School of Medicine Osaka University Suita Japan; ^7^ Department of Pathology of Mental Diseases National Institute of Mental Health, National Center of Neurology and Psychiatry Tokyo Japan

**Keywords:** clozapine, iPSC, monozygotic twins, neural development, neuronal differentiation, treatment‐resistant schizophrenia

## Abstract

Treatment‐resistant schizophrenia (TRS) affects 20%–30% of individuals diagnosed with schizophrenia and is effectively managed with clozapine. However, the molecular and cellular mechanisms that underlie its efficacy remain largely unclear. We previously generated induced pluripotent stem cell (iPSC) lines from a unique pair of monozygotic twins with TRS. One twin responded to clozapine (CLZ‐res), while the other did not (CLZ‐non‐res). Building on our previous study of these twins, we included healthy controls and focused on the early developmental stages of neuronal differentiation. To investigate the phenotypic differences in the developmental stages of neural cells between patient‐derived cells, we differentiated each iPSC line—from both patients and healthy individuals—into neural stem cells (NSCs) and subsequently induced the differentiation of NSCs into neurons. Our results demonstrated that NSCs derived from patients' iPSC lines exhibited impaired neuronal differentiation, with a more pronounced reduction in differentiation observed in CLZ‐non‐res cells than in CLZ‐res cells. RNA sequencing analysis revealed significant differences in the expression of genes involved in neuronal development between CLZ‐res and CLZ‐non‐res cells. These findings suggest that the differences in neuronal development may contribute to the variability in clozapine responsiveness. Although this study is limited to a single twin pair, this unique human model provides valuable insights into the molecular and cellular mechanisms underlying differential clozapine responses, offering a promising framework for the development of effective treatments for patients with TRS.

## Abstract 2

We investigated early neuronal development using iPSCs from twins with treatment‐resistant schizophrenia discordant for clozapine response. The non‐responder exhibited more pronounced impairment in differentiation than the responder. Transcriptomic analysis revealed distinct developmental gene expression profiles, suggesting that early neurodevelopmental defects may underlie the discordance in clozapine efficacy.

## Graphical abstract

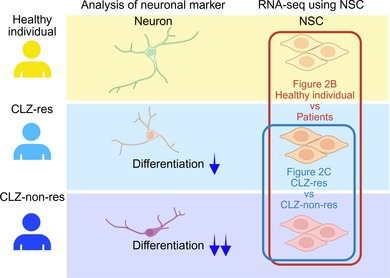

## 1 Introduction

Schizophrenia is a severe psychiatric condition with a prevalence of approximately 1% [1]. Despite the availability of both first‐ and second‐generation antipsychotics, a substantial proportion of individuals with schizophrenia—estimated at approximately 20%–30%—show little to no improvement, even after trials of two or more medications at appropriate doses. These individuals are considered to have treatment‐resistant forms of the disorder [2]. Although treatment‐resistant schizophrenia (TRS) imposes significant societal and economic burdens, the molecular and cellular mechanisms underlying this condition remain largely unclear. Clozapine is the most effective drug for TRS [2, 3]. While serotonergic and muscarinic properties of clozapine are thought to be important [3, 4, 5, 6], the specific biological mechanisms through which clozapine exerts its clinical effects are not yet fully understood. Given the substantial inter‐individual variability in responses to clozapine and its serious side effects, identifying the mechanisms underlying the therapeutic effects of clozapine, along with potential predictors of treatment response, is essential for advancing the understanding and management of TRS.

Modeling schizophrenia using induced pluripotent stem cell (iPSC) lines represents a promising strategy to explore the mechanistic basis of this complex condition [7]. Previously, we generated iPSC lines from a pair of monozygotic twins diagnosed with TRS, one of whom responded well to clozapine, while the other twin did not [8]. Monozygotic twin cases with TRS and discordant responses to clozapine are extremely rare, and to the best of our knowledge, this case is unique in the existing literature. Using differentiated neurons from the iPSC lines, several homophilic cell adhesion genes, such as protocadherins, exhibited differential expression between cells from the two patients.

In this study, we aimed to further investigate the phenotypic differences in the developmental stages of neural cells between patient‐derived cells. To achieve this, we analyzed the phenotypes of neural stem cells (NSCs) differentiated from iPSC lines derived from twin cases and healthy individuals. Our results demonstrated that patient‐derived NSCs exhibited impaired neuronal differentiation compared with those from healthy individuals, with substantial differences in the extent of neuronal differentiation observed between the patient‐derived lines.

## 2 Methods (Summary)

Neurons were generated from NSCs derived from iPSC lines of monozygotic twin cases with TRS and discordant responses to clozapine, as well as from healthy individuals. Differentiated neurons were subjected to immunocytochemistry to assess neuronal differentiation. Total RNA was isolated and subjected to reverse transcription‐quantitative PCR (RT‐qPCR) and RNA sequencing to examine gene expression profiles. Gene ontology (GO) analysis was subsequently performed to assess differences in RNA expression among neurons derived from each individual. Details are provided in the Supporting Information. Primer sequences are provided in Table S1.

## 3 Results

**FIGURE 1:**
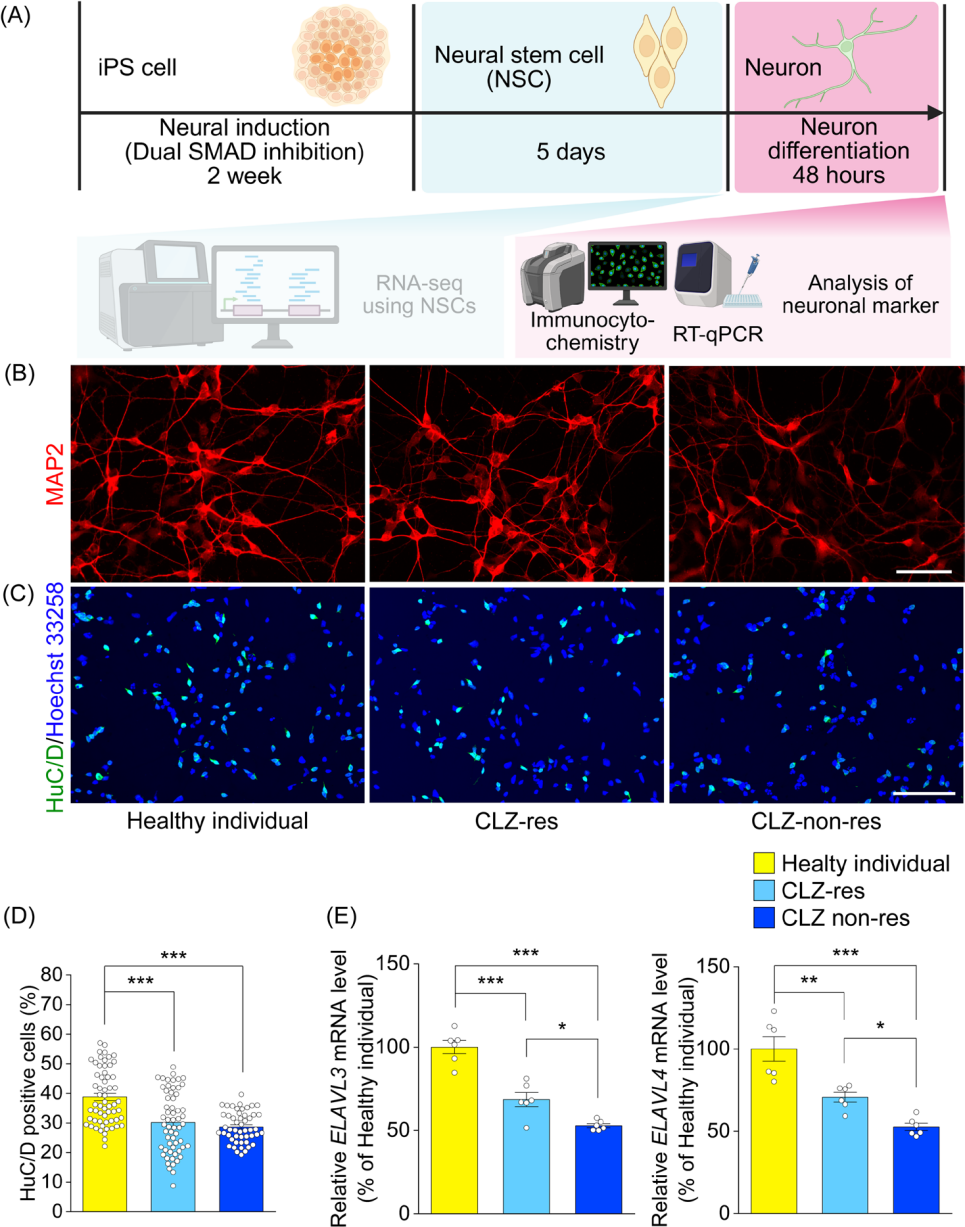
NSCs derived from twin patients exhibit impaired differentiation into neurons. (A) Time course of neuronal differentiation from iPSC lines into neurons via NSCs. (B) Representative images of immunostaining for MAP2. Scale bar, 50 μm. (C) Representative images of immunostaining for HuC/D, a neuronal marker. Scale bar, 100 μm. (D) Quantification of HuC/D‐positive cells. Healthy individual, *n* = 60; CLZ‐res, *n* = 60; CLZ‐non‐res, *n* = 50. (E) Relative mRNA levels of *ELAVL3* (left) and *ELAVL4* (right). Each *n* = 6. Data are presented as the mean ± SEM. Statistical analysis was performed using Tukey's multiple comparison test. *p < 0.05, **p < 0.01, ***p < 0.001. Illustrations were created using BioRender.com.

To further investigate the phenotypic differences in the developmental stages of neural cells among patient‐derived cells, we differentiated each iPSC line from patients and healthy individuals into NSCs. These NSCs were induced to differentiate into neurons (Figure 1A). Representative immunofluorescence images of neurons stained with MAP2 are shown in Figure 1B. Neuronal differentiation was quantified by immunostaining for the neuron‐specific RNA‐binding proteins Hu antigen C (HuC) and Hu antigen D (HuD). The proportion of HuC/HuD‐positive neurons was significantly lower in the differentiated cells from both clozapine‐responder (CLZ‐res) and clozapine‐non‐responder (CLZ‐non‐res) than in those from healthy individuals (Figure 1C,D). Furthermore, RT‐qPCR analysis demonstrated that the expression levels of *ELAVL3* (encoding HuC) and *ELAVL4* (encoding HuD) mRNA were lower in patient‐derived cells than in those from healthy individuals (Figure 1E), suggesting that neuronal differentiation from NSCs into neurons was impaired in patient‐derived cells. Interestingly, while no clear differences were observed in the immunocytochemical experiments, RT‐qPCR analysis demonstrated significant differences in the expression levels of *ELAVL3* and *ELAVL4* mRNA, with CLZ‐non‐res showing markedly lower expression than CLZ‐res (Figure 1E). Considering that no significant differences were detected by immunocytochemical analysis, RT‐qPCR analysis revealed distinct differences in the expression levels of *ELAVL3* and *ELAVL4*; these findings suggest that subtle, yet biologically relevant differences in neuronal differentiation may exist between the CLZ‐res and CLZ‐non‐res cells.

**FIGURE 2:**
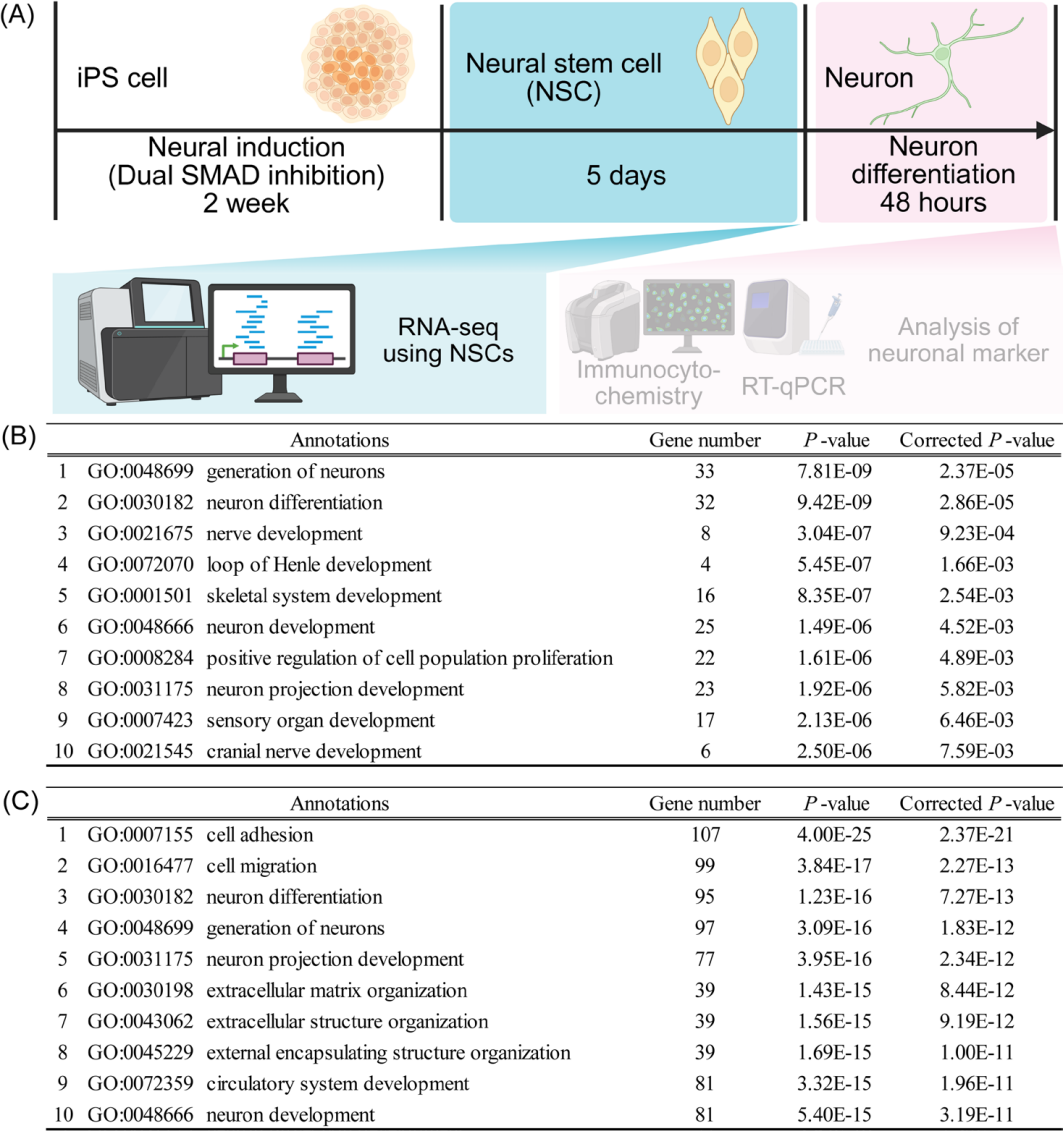
RNA sequencing analysis using NSCs. (A) Time course of RNA sequencing using NSCs. (B) GO terms enriched in the gene set differentially expressed between healthy individuals and patients (CLZ‐res and CLZ‐non‐res). (C) GO terms enriched in the gene set differentially expressed between CLZ‐res and CLZ‐non‐res. Illustrations were created using BioRender.com.

To investigate the molecular basis of the differences in neuronal differentiation observed between cells derived from patients and healthy individuals, we performed RNA sequencing on the NSC samples (Figure 2A). Genes with transcripts per million (TPM) ≥ 1 and |log_2_ fold change| ≥ 1 [Healthy individuals vs. patients (CLZ‐res and CLZ‐non‐res)] were extracted and analyzed using GO analysis. We found that 86 and 41 genes were upregulated and downregulated, respectively. These differentially expressed genes were subjected to unbiased GO analysis, and the top 10 enriched terms are shown in Figure 2B. Notably, most of these GO terms were associated with neuronal development. To validate the results of the RNA‐sequencing and GO enrichment analyses, we performed quantitative RT‐qPCR. Specifically, for the comparison between healthy individual‐ and patient‐derived cells, we examined the top 10 genes with the highest fold changes from each of the top‐ranking GO terms: *generation of neurons* (GO:0048699) and *neuron differentiation* (GO:0030182) (Figure S1). These results further support that neuronal differentiation is impaired in patient‐derived cells.

Next, genes with TPM ≥ 1 and |log_2_ fold change| ≥ 1 (CLZ‐res vs. CLZ‐non‐res) were extracted and analyzed using GO analysis. We found that 68 and 455 genes were upregulated and downregulated, respectively. These differentially expressed genes between CLZ‐res and CLZ‐non‐res were enriched in GO terms primarily associated with neural development (Figure 2C). These results further support the notion that biologically relevant differences in neuronal differentiation may exist between the CLZ‐res and CLZ‐non‐res cells. To validate the results of the RNA‐sequencing and GO enrichment analyses, we performed quantitative RT‐qPCR to examine the top 10 genes (or 9 for the upregulated set) with the highest fold changes from each of the top‐ranking GO terms: *neuron differentiation* (GO:0030182) and *generation of neurons* (GO:0048699) (Figure S2). Interestingly, the differentially expressed genes between CLZ‐res and CLZ‐non‐res cells were also enriched for the GO term “*cell adhesion* (GO:0007155)”, consistent with our previous findings in NGN2‐induced relatively mature neurons [8]. We further confirmed the expression changes of representative genes within the GO term “*cell adhesion* (GO:0007155)” using RT‐qPCR (Figure S3). This suggests that transcriptional dysregulation of cell‐adhesion‐related genes occurs in NSCs. Finally, we analyzed expression levels of NSC identity and neuronal subtypes genes and found that several genes were differentially expressed among cells derived from healthy individuals, CLZ‐res, and CLZ‐non‐res patients (Table S2).

## 4 Discussion

**FIGURE 3:**
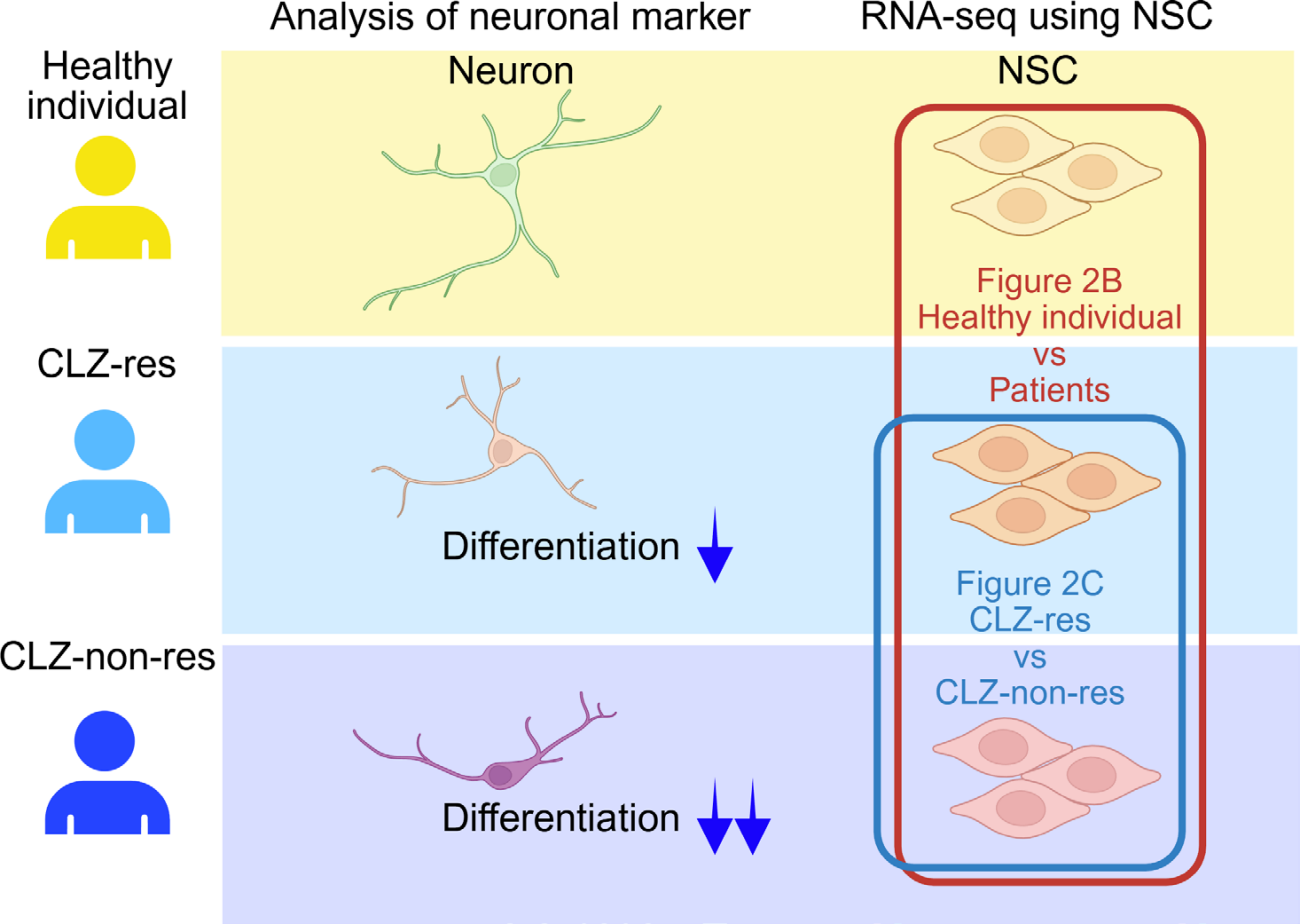
Impaired neuronal differentiation in patient NSCs, differing between CLZ‐res and non‐res cells.

In this study, we examined the characteristics of NSCs generated from iPSC lines derived from a pair of monozygotic twin cases with TRS, one of whom responded well to clozapine while the other did not, along with healthy individuals (Figure 3). Our analyses revealed that NSCs from patient‐derived cells exhibited impaired neuronal differentiation from NSCs into neurons compared with those from healthy individuals (Figure 1C–E). Notably, neuronal differentiation appeared to differ between the CLZ‐res and CLZ‐non‐res cells (Figures 1E and 2C), suggesting that the inter‐individual variability in clozapine response may already be present at the NSC stage. Furthermore, our findings suggest that alterations in neuronal lineage specification during the early stages of differentiation may contribute to the differential response to clozapine observed in the patients (Table S2).

To date, numerous studies have utilized iPSC technology to investigate schizophrenia [7, 9, 10, 11, 12]. However, our study is unique in that we used iPSC lines derived from a pair of monozygotic twin cases with TRS, one of whom twin responded well to clozapine, and the other did not. Given that monozygotic twins share nearly identical genetic backgrounds, neurons derived from their iPSC lines provide an ideal model for investigating the molecular and cellular basis of inter‐individual variability in clozapine response.

Using iPSC lines‐derived neural stem cells, we observed differential expression of genes involved in neural development and cell adhesion between the CLZ‐res and CLZ‐non‐res cells (Figure 2C). In our previous study, we identified widespread differences in DNA methylation between neurons derived from the CLZ‐res and CLZ‐non‐res cells [13]. These epigenetic variations may be present at the NSC stage and could underlie the differential expression of genes involved in neural development and cell adhesion, potentially leading to differences in neuronal differentiation between the CLZ‐res and CLZ‐non‐res. Alternatively, or in addition, although we did not identify genetic variation in exons [8], there may be differences in intronic variants between the patients.

While the precise molecular and cellular mechanisms underlying the clinical effects of clozapine have yet to be fully elucidated, clozapine acts on multiple neurotransmitter systems, including dopaminergic, serotonergic, muscarinic, adrenergic, cholinergic, and histaminergic pathways [3, 4]. We demonstrated that neuronal differentiation from NSCs into neurons differed between the CLZ‐res and CLZ‐non‐res cells, potentially leading to differences in synaptic functions across these various neurotransmitter systems. Additionally, the differential expression of cell adhesion molecules may also contribute to alterations in synaptic functions.

A limitation of this study is that the analysis was restricted to a single pair of monozygotic twins. Additional studies involving monozygotic twin cases with TRS and discordant responses to clozapine are needed to validate the current findings. However, such cases are extremely rare, making it generally very difficult to identify another pair of monozygotic twins with TRS and discordant responses to clozapine. A larger cohort of clozapine responders and non‐responders, rather than monozygotic twin cases, would be useful to further support the conclusions drawn from this study.

In the present study, we used iPSC‐based technology to identify possible molecular and cellular mechanisms behind inter‐individual variability in clozapine response. Our findings not only provide insight into how clozapine exerts its molecular and cellular effects but may also contribute to the development of new and more effective therapeutic strategies for patients with TRS.

## Author Contributions

Shotaro Kawano, Hitoshi Hashimoto, Ryota Hashimoto, and Takanobu Nakazawa conceived and designed the experiments, and wrote the manuscript. Shotaro Kawano, Sayaka Katayama, Masaya Ogawa, Rei Endo, Naoto Ikeda, Yuuri Ikeuchi, Tomoki Mita, Hikari Takei, Nanaka Gotoda‐Nishimura, Daiki Miura, Hotaka Fukushima, and Takanobu Nakazawa designed and performed the cellular experiments, and analyzed the data. Ryota Hashimoto recruited and characterized the patients. All authors read and approved the final version of the manuscript.

## Funding

This work was partly supported by the JSPS KAKENHI [grant numbers, JP23KJ1953 (Shotaro Kawano), JP23H00395 (Hitoshi Hashimoto), JP24K22022 (Hitoshi Hashimoto), and JP24K02182 (Takanobu Nakazawa)]; MEXT KAKENHI [grant number JP18H05416 (Hitoshi Hashimoto and Takanobu Nakazawa)]; AMED [grant numbers JP21dm0207117 (Hitoshi Hashimoto), JP25ama121052 (Hitoshi Hashimoto), JP25ama121054 (Hitoshi Hashimoto), JP24wn0425012 (Ryota Hashimoto, and Takanobu Nakazawa), JP25wm0625521 (Hitoshi Hashimoto, Ryota Hashimoto, and Takanobu Nakazawa), and JP19gm1310003 (Takanobu Nakazawa)]; Takeda Science Foundation, Japan (Hitoshi Hashimoto and Takanobu Nakazawa).

## Ethics Statement

The study was conducted with the approval of the Research Ethics Committees of Tokyo University of Agriculture, Osaka University, and the National Center of Neurology and Psychiatry. All procedures were carried out in accordance with the Declaration of Helsinki of the World Medical Association.

## Consent

Written informed consent was obtained from all participants.

## Conflicts of Interest

The authors declare no conflicts of interest.

## Supporting information


**Data S1:** npr270097‐sup‐0001‐Supinfo1.docx.

**Data S2:** npr270097‐sup‐0002‐Supinfo2.docx.

**Table S1:** Primer sequences used in this study.

**Table S2:** Expression levels of NSC identity and neuronal subtypes genes (TPM).

**Figure S1:** RT‐qPCR validation of top‐ranked genes identified in the comparison between healthy individual and patient‐derived cells. We performed quantitative RT‐qPCR for the top 10 genes selected from each of the top‐ranking GO terms: “generation of neurons” (GO:0048699) and “neuron differentiation” (GO:0030182). The top 10 genes selected were identical between the two GO terms. Data are presented as mean ± SEM. Statistical analysis was performed using Tukey's multiple comparison test. *p < 0.05, **p < 0.01, ***p < 0.001.

**Figure S2:** RT‐qPCR validation of top‐ranked genes identified in the comparison between the CLZ‐res and CLZ‐non‐res cells. We performed quantitative RT‐qPCR for the top 10 genes (or 9 for the upregulated set) selected from each of the top‐ranking GO terms: “neuron differentiation” (GO:0030182) and “generation of neurons” (GO:0048699). The top 10 genes selected were identical between the two GO terms. Data are presented as mean ± SEM. Statistical analysis was performed using Student's *t*‐tests. *p < 0.05, **p < 0.01, ***p < 0.001.

**Figure S3:** RT‐qPCR validation of top‐ranked genes identified in the comparison between the CLZ‐res and CLZ‐non‐res cells. We performed quantitative RT‐qPCR for the top 10 genes classified under the GO term “cell adhesion” (GO:0007155). Note that the RT‐qPCR data for *BMP6*, *ADGRB1*, *SPINK5*, *PRTG*, *CAV1*, *LGALS1* and *TGFB2* are identical to those presented in Figure S2. Data are presented as mean ± SEM. Statistical analysis was performed using Student's *t*‐tests. *p < 0.05, **p < 0.01, ***p < 0.001.

## Data Availability

The data are not publicly available due to privacy and ethical restrictions (i.e., we did not obtain informed consent for the public availability of raw data).
