# Supplementary material for: Differential Neuronal Development in iPSC‐Derived Neural Stem Cells From Monozygotic Twin Cases With Treatment‐Resistant Schizophrenia and Discordant Responses to Clozapine
Source: Neuropsychopharmacol Rep. 2026 Mar 22;46(2):e70097. doi: 10.1002/npr2.70097 (PMC13283898; doi:10.1002/npr2.70097)
Supplement: Supplementary file 1 — Data S1: npr270097‐sup‐0001‐Supinfo1.docx. [file NPR2-46-e70097-s007.docx]

**Supporting Information**

**Differential Neuronal Development in iPSC-Derived Neural Stem Cells from Monozygotic Twin Cases with Treatment-Resistant Schizophrenia and Discordant Responses to Clozapine**

Shotaro Kawano, Sayaka Katayama, Masaya Ogawa, Rei Endo, Naoto Ikeda, Yuuri Ikeuchi, Tomoki Mita, Hikari Takei, Nanaka Gotoda-Nishimura, Daiki Miura, Hotaka Fukushima, Hitoshi Hashimoto, Ryota Hashimoto, and Takanobu Nakazawa

**Detailed Methods**

*Generation of induced pluripotent stem cell (iPSC) lines*

All iPSC lines were generated following previously described methods [1, 2]. Briefly, iPSC lines were established from immortalized B cells derived from both healthy individuals and patients using a non-integrating episomal vector expressing OCT3/4, SOX2, KLF4, L-MYC, LIN28, dominant-negative TP53, and EBNA1. Written informed consent was obtained from all subjects. The subject information was as follows:

Patients (CLZ responder (P0070) and CLZ non-responder (P0052))

The patient-derived iPSC lines used in this study were the same as those described in our previous report [2]. Independent 2 lines (P0070, P0070-2B, 9B; P0052, P0052-1B, 4B) were used for analysis.

Healthy individuals

P1399–Healthy control-derived iPSC lines used in this study were the same as those described in our previous report [3].

P0431–The subject was a 60-year-old Japanese male.

*Culture of iPSC lines*

iPSCs were maintained on 6-well plates (353046, Corning, NY, USA) coated either with iMatrix-511 (892011, Nippi, Tokyo, Japan) at 0.5 μg/cm² or with Matrigel hESC-Qualified Matrix (354277, Corning) diluted 1:50 in D-MEM (High Glucose) with L-Glutamine (043-30085, Wako, Osaka, Japan) at 1 mL/well. Cells were seeded at a density of 1–6×10⁴ cells/well and passaged every 5–8 days. StemFit medium (AK02N, Ajinomoto, Tokyo, Japan) was used for cell maintenance, and 10 μM Y-27632 (034-24024, Wako) was added from the time of passaging until the following day. For passaging, cells were dissociated using a mixture of TrypLE Select solution [0.5 mM EDTA (15575-038, Thermo Fisher Scientific, CA, USA) in PBS and TrypLE Select enzyme (12563-011, Thermo Fisher Scientific) at a 1 : 1 ratio].

*Neural induction from iPSC lines*

Neural induction was performed using the dual SMAD inhibition method with PSC Neural Induction Medium (A1647801, Thermo Fisher Scientific). iPSCs were passaged onto Matrigel-coated 6-well plates at a density of 1×10⁶ cells/well. The day after seeding, the medium was replaced with 2 mL/well of PSC Neural Induction Medium, and the cultures were maintained for approximately one week. For the subsequent passages, cells were dissociated with Accutase (AT104, Innovative Cell Technologies, CA, USA) and replated at 1×10⁶ cells/well in Neural Expansion Medium, which consisted of Neurobasal Medium (21103-049, Thermo Fisher Scientific), Advanced DMEM/F-12 (12634-028, Thermo Fisher Scientific), and Neural Induction Supplement (included with PSC Neural Induction Medium) mixed at a 49 : 49 : 2 ratio. The culture medium was supplemented with 10 μM of the ROCK inhibitor Y-27632 (034-24024, Wako) from the time of passaging until the following day. After approximately one week of culture in this medium, neural stem cells were obtained and used for the subsequent experiments.

*Neuronal differentiation from neural stem cells*

For neuronal differentiation, 12-well plates (353043, Corning) were first coated with Poly-L-ornithine (P3655, Sigma-Aldrich, MO, USA), followed by incubation with PBS containing 6.67 μg/mL human fibronectin (33016-015, Thermo Fisher Scientific) and 6.67 μg/mL mouse laminin (23017-015, Thermo Fisher Scientific) at 1 mL/well. Neural stem cells in culture were dissociated with Accutase and plated at a density of 6×10⁵ cells/well in neuronal differentiation medium. The neuronal differentiation medium consisted of BrainPhys Neuronal Medium (ST-05790, STEMCELL Technologies, BC, Canada), supplemented with 1% N2 supplement (141-09041, Wako), 2% B-27 supplement (17504-044, Thermo Fisher Scientific), 200 μM L-ascorbic acid (A4403, Sigma-Aldrich), 1 mM bucladesine sodium (029-16383, Wako), 20 ng/mL recombinant human BDNF (248-BD/CF, R&D Systems, MN, USA), 20 ng/mL recombinant human GDNF (212-GD/CF, R&D Systems), 500 ng/mL mouse laminin, and 1 μM DAPT (043-33581, Wako).

*Immunocytochemistry*

Neurons cultured on cover glasses (12 mm No.1-S, Matsunami Glass, Osaka, Japan) coated with Poly-L-ornithine, mouse laminin, and human fibronectin (as described above) were washed with PBS and fixed with 4% paraformaldehyde. After fixation, the cells were permeabilized with 0.1% Triton X-100 (T9284, Sigma-Aldrich) and blocked with 4% goat serum (16210-064, Thermo Fisher Scientific). The primary antibody [mouse anti-HuC/HuD (A-21271, Thermo Fisher Scientific; 1:100) and rabbit anti-MAP2 (AB5622-I, Sigma-Aldrich; 1:500)] was applied overnight at 4 °C, followed by incubation with the secondary antibody [Alexa Fluor 488-AffiniPure donkey Anti-mouse IgG (H+L) (715-545-150, Jackson ImmunoResearch Laboratories, PA, USA; 1:250) and goat anti-rabbit IgG (H+L) highly cross-adsorbed secondary antibody, Alexa Fluor 555 (A-21429, Thermo Fisher Scientific; 1:250)] for 2 h at room temperature. Nuclei were counterstained with Hoechst 33258 dye (382061-S, Merck Millipore, CA, USA; 1:250). Samples were mounted using Fluoromount (K024, Diagnostic BioSystems, CA, USA). Fluorescent imaging was performed using a BZ-X810 microscope (KEYENCE, Tokyo, Japan), and image analysis was conducted using the BZ-X800 Analyzer software (KEYENCE).

*Reverse transcript quantitative polymerase chain reaction (RT-qPCR)*

Total RNA was extracted from the cultured cells using the PureLink RNA Mini Kit (12183025, Thermo Fisher Scientific). Reverse transcription was performed with SuperScript III Reverse Transcriptase (18080085, Thermo Fisher Scientific) to generate cDNA. Quantitative real-time PCR was conducted using TB Green Premix Ex Taq™ II (Tli RNaseH Plus) (RR820D, Takara Bio, Shiga, Japan) and a real-time PCR detection system (CFX96, Bio-Rad Laboratories, CA, USA). Gene expression levels were quantified using the ΔΔCt method, with each sample run in technical triplicates and normalized to the expression of *ACTB*. Primer sequences are provided in Table S1.

*RNA sequencing*

Total RNA extracted from NSCs was sequenced using the HiSeq 2500 platform (Illumina, CA, USA). Gene expression levels were quantified as transcripts per million (TPM). *P*-values were corrected using the Bonferroni method and ranked by significance. The top 10 enriched terms were reported.

*Gene ontology analysis*

Gene Ontology (GO) analysis was performed using the ToppGene Suite (https://toppgene.cchmc.org/). Genes with TPM ≥ 1.0 and |log2 fold change| ≥ 1 were included in the analysis. The Biological Process category was used, with gene annotation size restricted to 1 ≤ n ≤ 2000.

*Data analysis and statistics*

Quantified data were analyzed using Tukey’s multiple comparison test or Student’s *t*-test. Statistical analysis was performed using the BellCurve for Excel (Social Survey Research Information, Tokyo, Japan).

**References**

1. Fujimori K., Tezuka T., Ishiura H., et al. Modeling neurological diseases with induced pluripotent cells reprogrammed from immortalized lymphoblastoid cell lines. *Mol Brain*. 2016;9(1):88.

2. Nakazawa T., Kikuchi M., Ishikawa M., et al. Differential gene expression profiles in neurons generated from lymphoblastoid B-cell line-derived iPS cells from monozygotic twin cases with treatment-resistant schizophrenia and discordant responses to clozapine. *Schizophr Res*. 2017;181:75-82.

3. Matsumura K., Seiriki K., Okada S., et al. Pathogenic POGZ mutation causes impaired cortical development and reversible autism-like phenotypes. *Nat Commun*. 2020;11(1):859.
