# Supplementary material for: Differential Neuronal Development in iPSC‐Derived Neural Stem Cells From Monozygotic Twin Cases With Treatment‐Resistant Schizophrenia and Discordant Responses to Clozapine
Source: Neuropsychopharmacol Rep. 2026 Mar 22;46(2):e70097. doi: 10.1002/npr2.70097 (PMC13283898; doi:10.1002/npr2.70097)
Supplement: Supplementary file 2 — Data S2: npr270097‐sup‐0002‐Supinfo2.docx. [file NPR2-46-e70097-s003.docx]

*tweetable abstract or social media opportunity*

We modeled treatment-resistant #Schizophrenia using iPSCs from monozygotic twins with discordant clozapine responses. Our study reveals that impaired early neuronal differentiation and gene expression may correlate with poor clinical response to clozapine. #Neuroscience
