# Supplementary figures and images for: Differential Neuronal Development in iPSC‐Derived Neural Stem Cells From Monozygotic Twin Cases With Treatment‐Resistant Schizophrenia and Discordant Responses to Clozapine
Source: Neuropsychopharmacol Rep. 2026 Mar 22;46(2):e70097. doi: 10.1002/npr2.70097 (PMC13283898; doi:10.1002/npr2.70097)

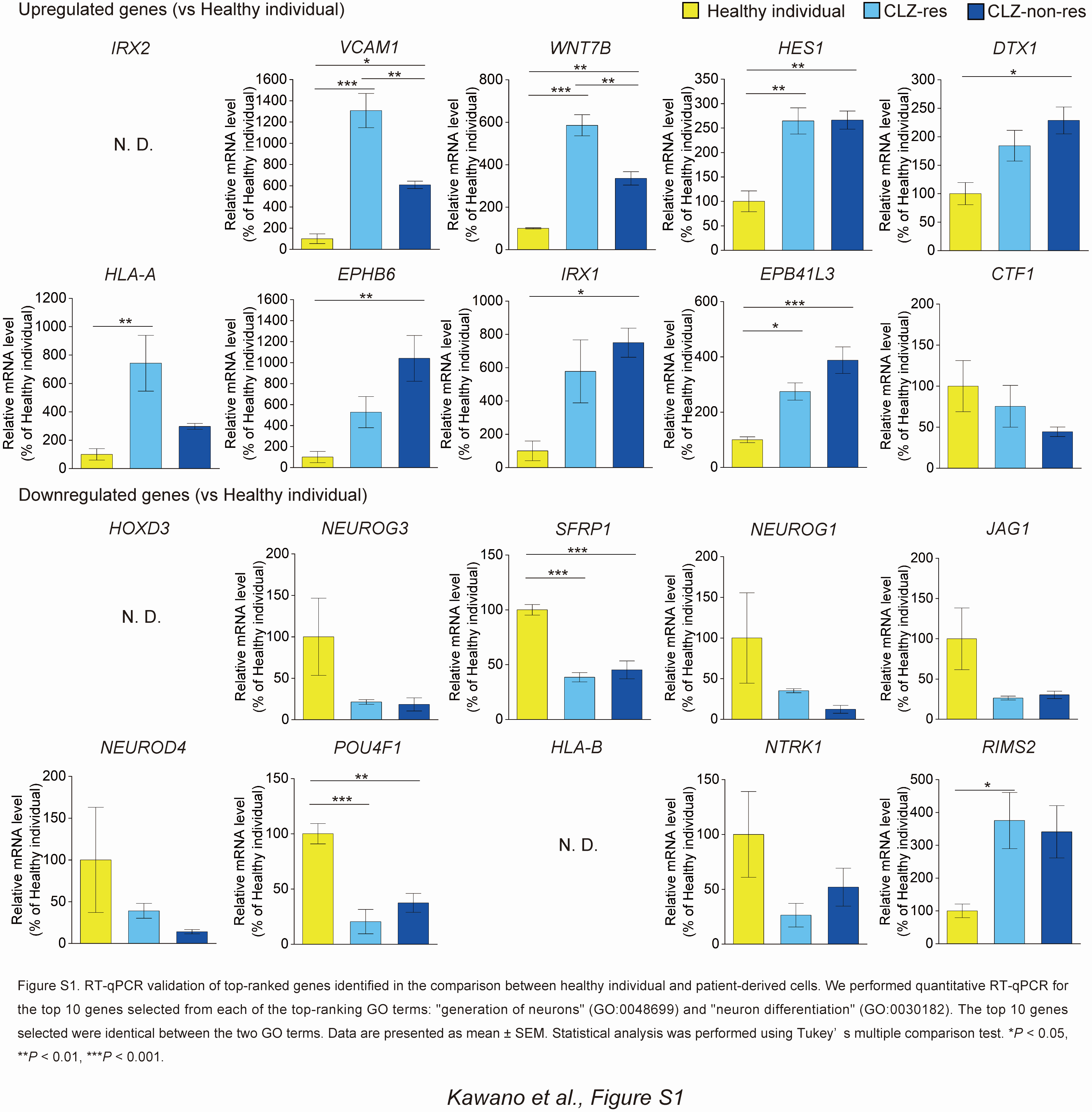

Supplement: Supplementary file 5 — Figure S1: RT‐qPCR validation of top‐ranked genes identified in the comparison between healthy individual and patient‐derived cells. We performed quantitative RT‐qPCR for the top 10 genes selected from each of the top‐ranking GO terms: “generation of neurons” (GO:0048699) and “neuron differentiation” (GO:0030182). The top 10 genes selected were identical between the two GO terms. Data are presented as mean ± SEM. Statistical analysis was performed using Tukey's multiple comparison test. *p < 0.05, **p < 0.01, ***p < 0.001. [file NPR2-46-e70097-s004.tiff]

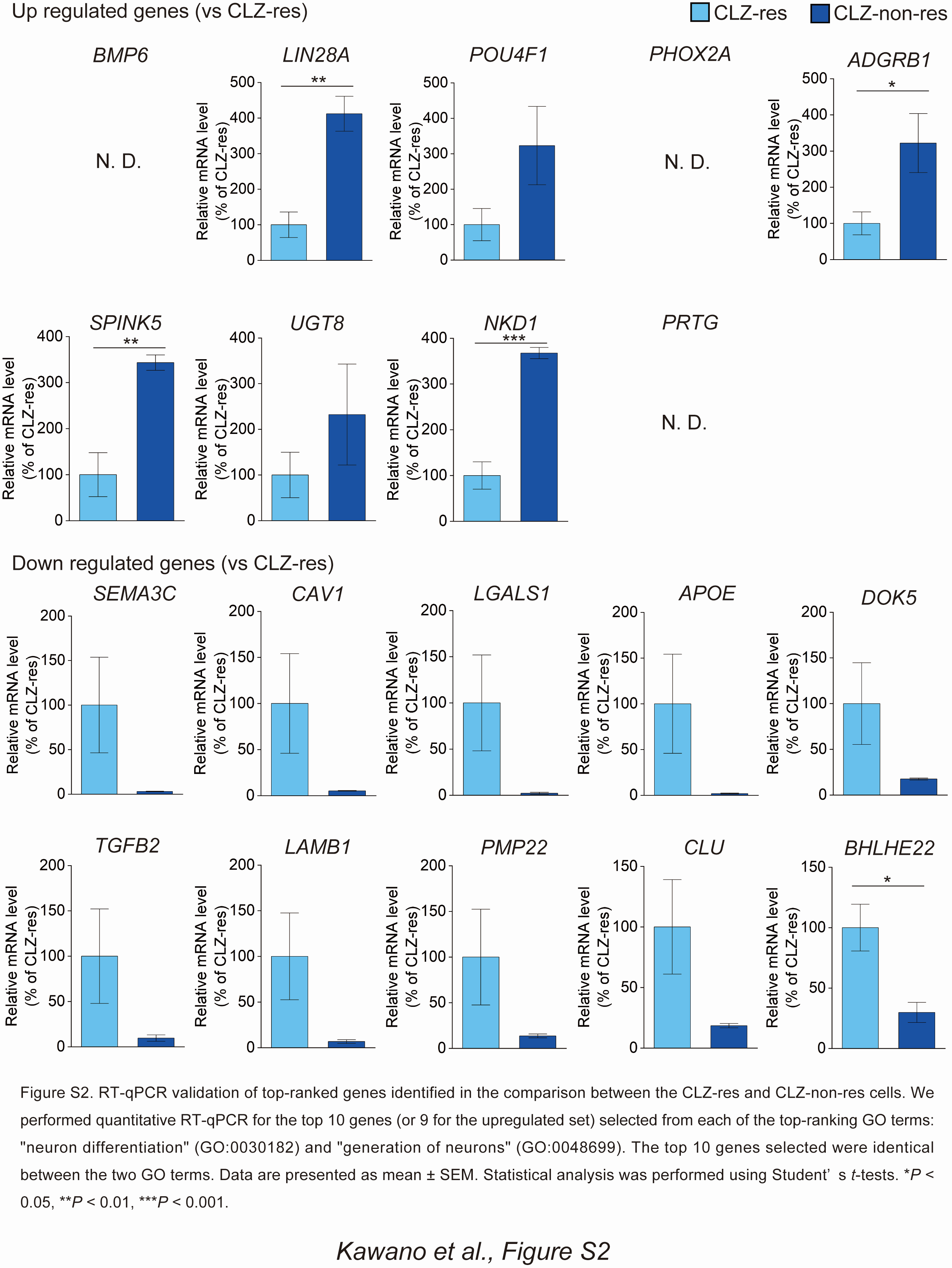

Supplement: Supplementary file 6 — Figure S2: RT‐qPCR validation of top‐ranked genes identified in the comparison between the CLZ‐res and CLZ‐non‐res cells. We performed quantitative RT‐qPCR for the top 10 genes (or 9 for the upregulated set) selected from each of the top‐ranking GO terms: “neuron differentiation” (GO:0030182) and “generation of neurons” (GO:0048699). The top 10 genes selected were identical between the two GO terms. Data are presented as mean ± SEM. Statistical analysis was performed using Student's t‐tests. *p < 0.05, **p < 0.01, ***p < 0.001. [file NPR2-46-e70097-s001.tiff]

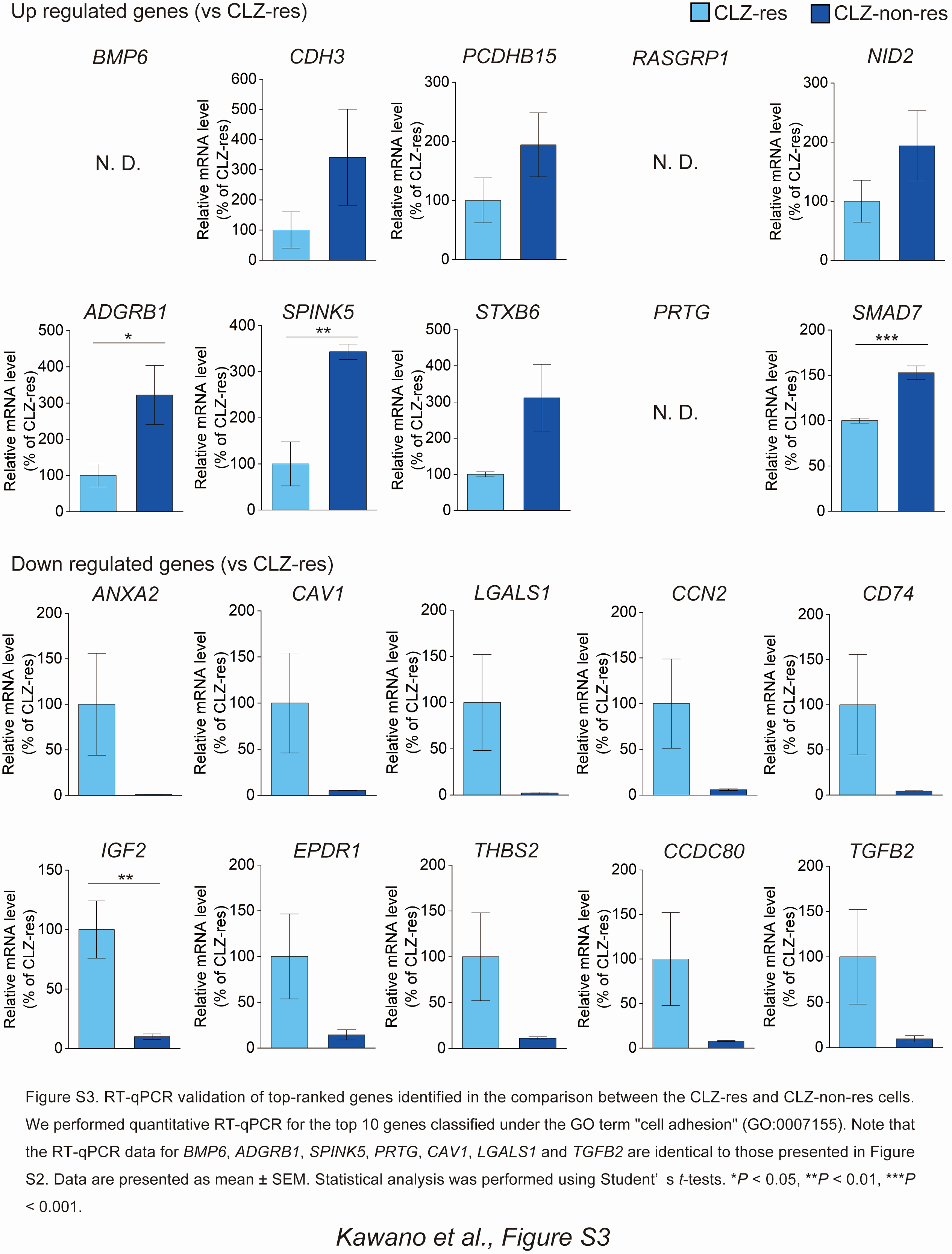

Supplement: Supplementary file 7 — Figure S3: RT‐qPCR validation of top‐ranked genes identified in the comparison between the CLZ‐res and CLZ‐non‐res cells. We performed quantitative RT‐qPCR for the top 10 genes classified under the GO term “cell adhesion” (GO:0007155). Note that the RT‐qPCR data for BMP6, ADGRB1, SPINK5, PRTG, CAV1, LGALS1 and TGFB2 are identical to those presented in Figure S2. Data are presented as mean ± SEM. Statistical analysis was performed using Student's t‐tests. *p < 0.05, **p < 0.01, ***p < 0.001. [file NPR2-46-e70097-s006.tiff]
